# Use of continuous subcutaneous insulin infusion in children and adolescents with type 1 diabetes mellitus: a systematic mapping review

**DOI:** 10.1186/s12902-022-00950-7

**Published:** 2022-02-19

**Authors:** Carolina Spinelli Alvarenga, Rebecca Ortiz La Banca, Rhyquelle Rhibna Neris, Valéria de Cássia Sparapani, Miguel Fuentealba-Torres, Denisse Cartagena-Ramos, Camila Lima Leal, Marcos Venicio Esper, Lucila Castanheira Nascimento

**Affiliations:** 1 Public Health Nursing Graduate Program, University of São Paulo at Ribeirão Preto College of Nursing, Ribeirão Preto, SP Brazil; 2 Joslin Diabetes Center, Harvard Medical School, Boston, MA USA; 3 Federal University of Santa Catarina, Florianopólis, Santa Catarina Brazil; 4 Universidad de los Andes, Santiago, Chile; 5 Universidad Andrés Bello, Santiago, Chile; 6 Interunit Doctoral Program in Nursing, University of São Paulo College of Nursing and the University of São Paulo at Ribeirão Preto College of Nursing, Ribeirão Preto, SP Brazil; 7 Maternal-Infant and Public Health Nursing Department, University of São Paulo at Ribeirão Preto College of Nursing, PAHO/WHO Collaborating Centre for Nursing Research Development, 3900 Av. Bandeirantes, Campus Universitário - Bairro Monte Alegre, Ribeirão Preto, São Paulo 14040-902 Brazil

**Keywords:** Child, Adolescent, Diabetes mellitus, type 1, Insulin infusion systems, Review, Pediatric nursing

## Abstract

**Background:**

Among the treatments for type 1 diabetes mellitus (T1DM), Continuous Subcutaneous Insulin Infusion (CSII) is a device that infuses insulin through the subcutaneous tissue in an uninterrupted manner and that comes closest to the physiological secretion of insulin. The use of CSII can provide the family with greater security and children and adolescents have more autonomy in relation to the treatment of T1DM. There is a lack of reviews that systematically gather the mounting evidence about the use of CSII in children and adolescents with T1DM. Therefore, the aim of this review was to group and describe primary and secondary studies on the use of CSII in children and adolescents with T1DM.

**Methods:**

A systematic mapping review was performed based on searches in the following databases: PubMed, Embase, CINAHL, Lilacs and PsycINFO, using a combination of descriptors and keywords. The screening of the studies was carried out with the aid of the Rayyan software and reading in full was conducted independently by two reviewers. The data extraction of the studies was performed using an extraction tool adapted and validated by researchers specialized in diabetes. The data were analyzed according to the content analysis technique. The map from geocoding of the studies was produced using the ArcGis 10.5 software.

**Results:**

A total of 113 studies were included in the review, including primary studies, literature reviews and gray literature publications. The content analysis of the results of the studies allowed for the identification of four categories: 1) metabolic control; 2) support networks; 3) benefits of using CSII; and 4) challenges of using CSII, each category having its respective subcategories. The review also made it possible to conduct a rigorous mapping of the literature on the use of CSII considering the location of development and the design of the studies.

**Conclusions:**

The use of CSII should be indicated by health professionals able to prepare children, adolescents, and their families for the treatment of T1DM, and, despite being a technological device, it may not be suitable for the entire pediatric population.

**Supplementary Information:**

The online version contains supplementary material available at 10.1186/s12902-022-00950-7.

## Background

Annually, about 128,900 children and adolescents are diagnosed with Type 1 Diabetes Mellitus (T1DM) [1]. Global estimates for 2019 were that around 1,110,100 children and adolescents were diagnosed with T1DM [1]. Drug treatment of T1DM consists of insulin injections, which can be administered by syringe, insulin pen or Continuous Subcutaneous Insulin Infusion (CSII) systems, so called insulin pumps [2, 3]. CSII is a device that continuously infuses insulin through the subcutaneous tissue [2]. This is the therapy that most closely resembles physiological insulin secretion, releasing minimum doses of insulin continuously (basal infusion) and one-off doses at prandial times or to correct hyperglycemia (bolus infusion) [4, 5].

In addition to the demands related to drug treatment, the diagnosis of diabetes influences the family functioning, affecting the educational, emotional, behavioral and nutritional development of the child [6]. The care to control the disease that must be performed daily consists of glycemic monitoring, drug treatment, carbohydrate counting, and physical exercise, in addition to maintaining good eating habits [7, 8]. The use of technologies, such as CSII, provides the family with greater security, so that children and adolescents develop more autonomy in relation to the treatment of T1DM [9]. CSII has been used successfully in pediatrics and provides several benefits to children and their families [2].

The first publications on the use of CSII in children and adolescents with T1DM date from 1979 [10, 11]. The available literature on this topic is gathered from systematic reviews [12–14], narratives [15–32] and discussions [33–37]. However, no systematic mapping review published in the scientific literature related to the topic was identified. Developing a systematic mapping of the scientific knowledge produced on the use of CSII in children and adolescents with T1DM would facilitate the synthesis of knowledge concerning all the literature produced to date, regardless of the methodology used in the published studies. This review will make it possible to identify gaps that has not yet been addressed and require future research. In addition, reviews are important tools to guide the construction of health policies and to develop implications for clinical practice [38]. Therefore, the purpose of this review was to group and describe existing evidence on the use of CSII in children and adolescents with T1DM.

## Method

### Design

This is a systematic mapping review, which is characterized by the integration of qualitative and quantitative studies, review studies and gray literature publications, and provides a comprehensive representation of the available literature on a specific topic [38–40]. For the systematic development of this review, the six steps proposed by James, Randall and Haddaway [38] were followed: 1) Development of the review protocol; 2) Search for evidence; 3) Screening of evidence; 4) Codification; 5) Critical evaluation (optional); and 6) Description and visualization of the findings [38].

### Search for evidence

The identification of eligible studies was carried out independently by two reviewers who systematically searched the literature in five databases: PubMed, CINAHL, PsycINFO, Embase and LILACS. The search strategy was developed using the PCO tool (P: Population; C: Context; O: Outcome) [41] and based on the following research question: “What studies have been produced on the use of CSII with children and adolescents with T1DM?”

For the construction of the search strategy, controlled descriptors from MeSH, CINAHL subject headings, APA Thesaurus, Emtree and DECs were used, combined with keywords and the Boolean operators AND, OR and NOT. In addition, studies were manually selected and included by unsystematic searches in the reference lists of studies included in the review. In accordance with the language fluency of the researchers, the search was limited to studies in English, Portuguese and Spanish. To capture the largest possible number of published studies on the subject, an initial time filter was not added and the final time limit was December 2020.

### Inclusion and exclusion criteria

Primary and secondary, quantitative and qualitative studies were included, which presented in their objectives aspects about the use of CSII in children and adolescents (0-18 years) with T1DM. The study could include the perspectives of the children and adolescents themselves, family members, and health professionals. Studies that were excluded were: those comparing multiple dose insulin injection therapy with therapy using CSII; that included young adults and adults together in the samples; patients with type 2 diabetes mellitus (T2DM); and that included CSII users with a focus on the continuous glucose monitoring system (CGM) or artificial pancreas.

### Screening of evidence

A total of 2088 files were recovered, 2091 of them through the systematic search in the databases and three after analysis of the references of the included studies. After excluding 266 duplicate publications, a total of 1825 titles and abstracts were independently screened by the two reviewers according to the eligibility criteria. A Kappa index of 0.87 was obtained, equivalent to an almost perfect interobserver agreement [42]. This process was achieved with the aid of the Rayyan QCRI software [43].

**Fig. 1:**
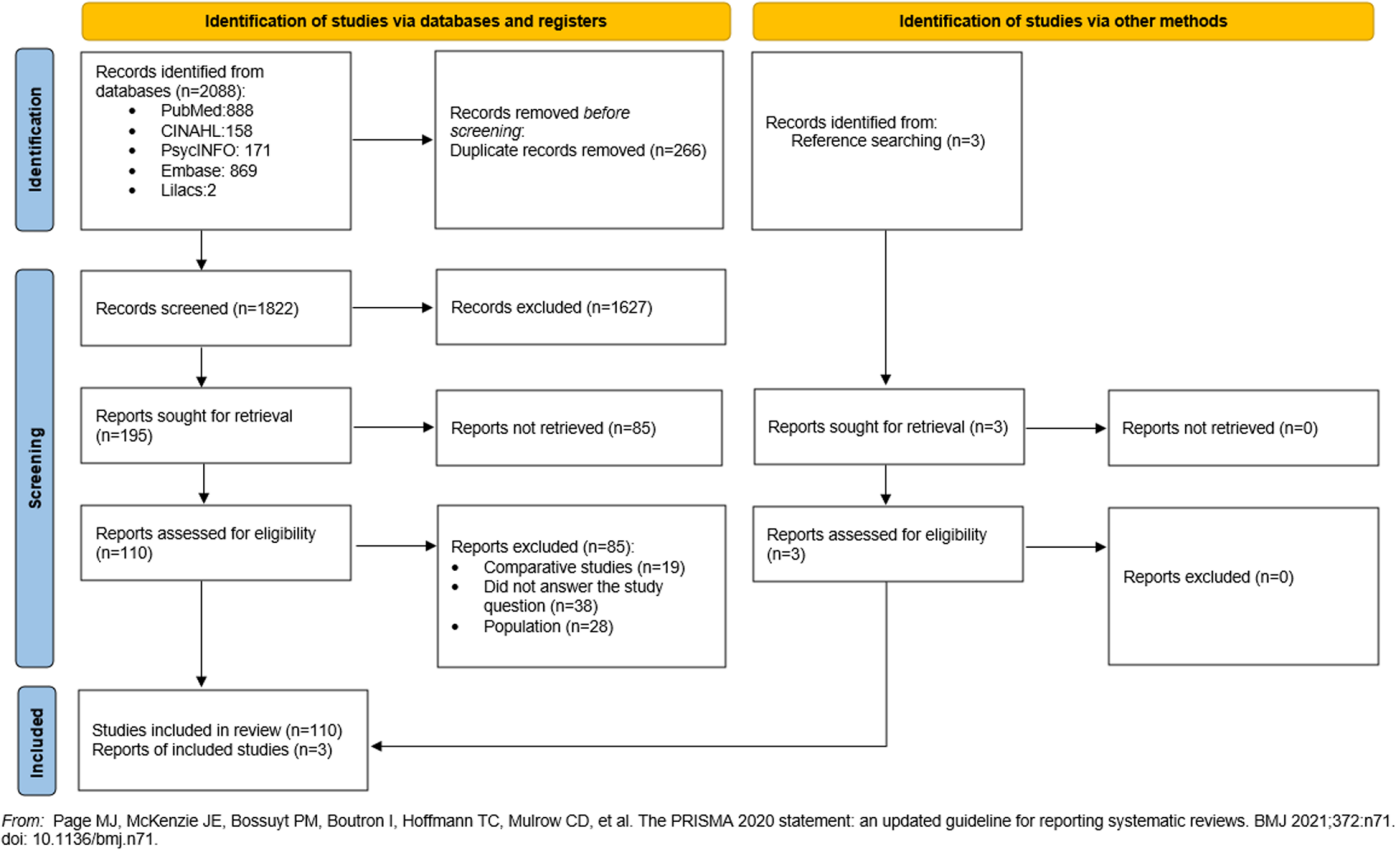
PRISMA flow diagram [45]

In the eligibility stage, 198 publications were selected for reading in full by two independent reviewers (CSA; CLL). In cases of disagreement between them, a third reviewer (LCN) was consulted [44]. Of the 198 publications, 85 were excluded after reading in full, as they did not meet the inclusion criteria of the review. The final sample consisted of 113 references. The screening process of the studies is illustrated by means of the flow diagram of the Preferred Reporting Items for Systematic and Meta-Analyzes (PRISMA), as shown in Fig. 1 [45].

### Codification

Data extraction was carried out in stage four of the development of the systematic mapping review, using a data extraction tool adapted from the National Health Service Center for reviews and dissemination [46]. This tool was submitted to content validation by three researchers (ROLB, VCS and LCN), and nurses qualified at the doctorate and postdoctoral level (LCN and ROLB), two of them specialists in T1DM (ROLB and VCS).

In this validation, the relevance of each item to be extracted was evaluated, in order to answer the research question of this mapping review. Adjustments were made and, at the end of the content validation, the tool included the following information that indicated the variables to be extracted from the studies included in the review: study title, authors, year of publication, type of study (document), design, journal of publication, language, study location, objectives, search period (in the case of secondary studies), duration of the study, population, sampling strategy, intervention, and methods and results. Additionally, to assist in the organization of the data extraction process, the studies were separated according to thematic similarity, objectives, and design.

The following information was extracted from the results of the included studies: response of clinical indicators in the use of CSII (for example, variation of HbA1c, BMI, level of hypoglycemia and episodes of diabetic ketoacidosis); influence of the use of CSII on the psychological, social and emotional factors of children, adolescents and their families; effectiveness of using CSII; challenges associated with the use of CSII; and directions for using CSII. The extraction step was carried out independently by the main researcher and another member of the research group. After the initial extraction, three researchers (ROLB; LCN; RRN) verified the data collected by conferring with the original studies.

Subsequently, the extracted data were subjected to content analysis [47], which went through the following steps: 1) Codification of the extracted data, in which there was an exhaustive reading to identify significant words, passages and categories. Notes were written in the margins to describe all aspects of the content of interest contained in the data; 2) At this stage, the process of categorizing and organizing the codes raised earlier began, considering the affinity, variability and range of the codes, relating them to the composition of the categories. It is worth mentioning that, at this stage, the same code could belong to two different categories, due to the possibility of providing different information to be analyzed; 3) In the third stage, known as integration, the categories were integrated into larger themes, in order to present the contributions of studies in relation to the use of CSII in children and adolescents with T1DM.

### Production of the systematic map

To illustrate the geographical location in which the included studies were developed, a spatial mapping was carried out, in which it is possible to visualize the categories of the country and the type of study. This procedure required the geocoding of the locations where the studies were developed, by considering the location of the corresponding author for the analysis.

We opted for the production of a figure for the spatial mapping of the production of scientific studies focused on the use of CSII, due to its potential to identify the world regions where the studies are concentrated on this theme, as well as showing those that need more research on the theme addressed in this review. The geographic coordinates of the addresses were obtained by Google maps and, later, the study sites were geocoded by the ArcGis 10.5 software. The locations of the geocoded studies followed the Universal Transverse Mercator (UTM) and the Geocentric Reference System for the Americas (SIRGAS) 2000 projection [48].

## Results

**Fig. 2:**
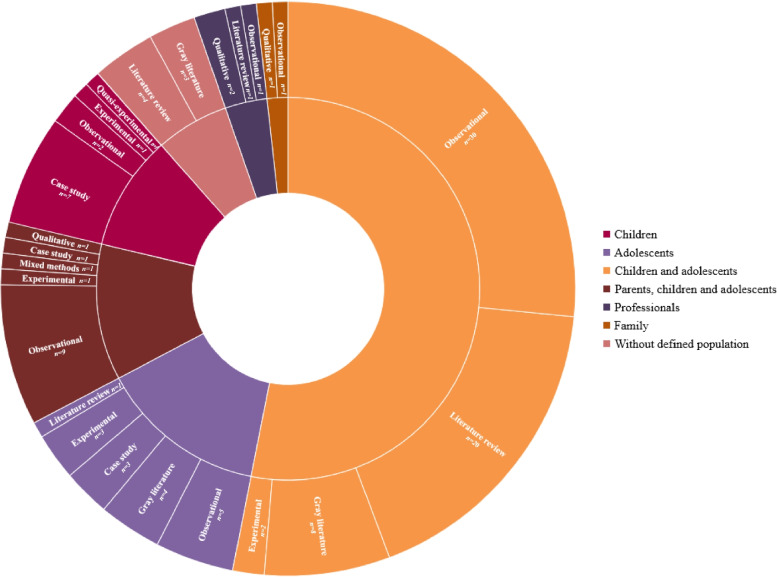
Studies included according to methodological design and studied population

**Fig. 3:**
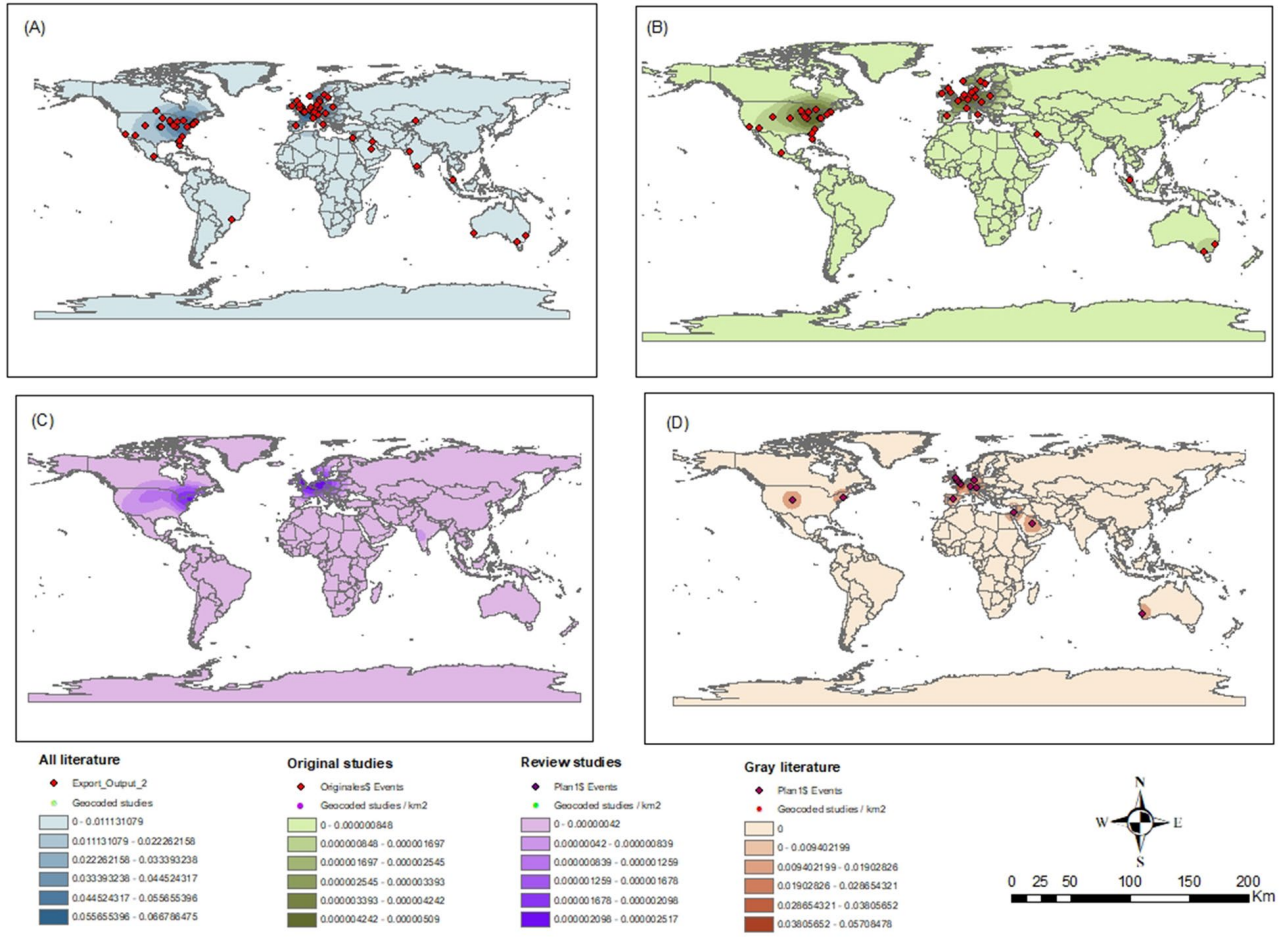
The geocoding of the sites of development of the analyzed studies

Of the total of 113 studies included in this review, there was a higher frequency of publications in the last decade, with 59 studies published between 2010 and 2020 compared to 40 in the period from 2000 to 2009. The frequency of studies prior to the year 2000 totaled 14 studies. In Fig. 2, the number of studies included in the review is illustrated according to the methodological outline and the population studied. Figure 3 illustrates the geocoding of the sites of development of the analyzed studies.

**Fig. 4:**
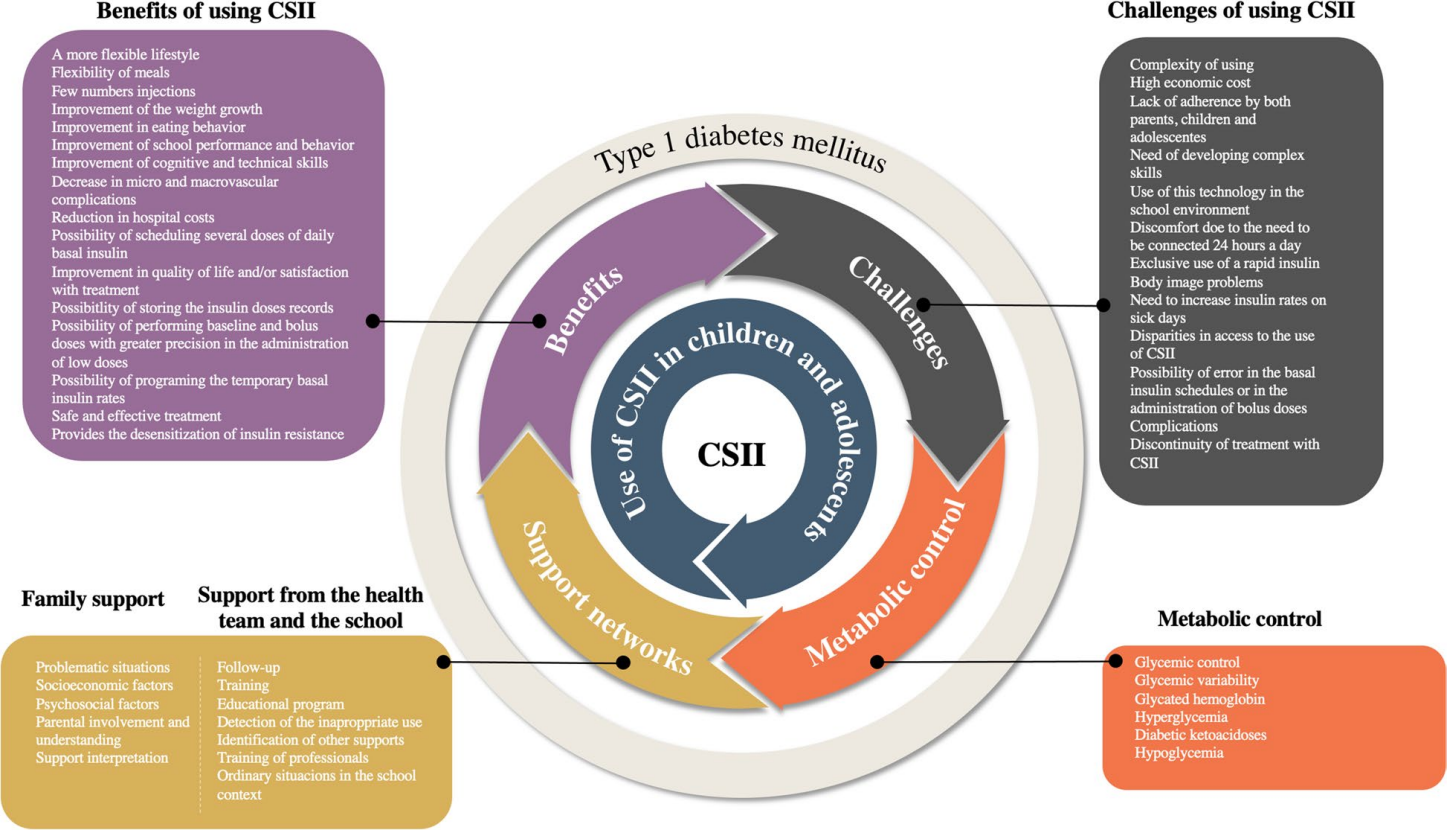
Systematic map of the categories and subcategories formed after analyzing the studies

The content analysis of the results of the studies included in this review allowed for the construction of the following categories: metabolic control, support networks, benefits of using CSII, and challenges of using CSII. Figure 4 presents a systematic map with the categories and subcategories formed after the analysis.

### Metabolic control

This category presents included studies that described the metabolic parameters during the use of CSII in children and adolescents with T1DM.

Forty-two studies addressed in their results that the use of CSII led to an improvement in glycemic control [10, 13, 14, 16, 18, 20, 22, 25, 27, 32, 34, 49–79] and seven reported a decrease in glycemic variability [14, 22, 28, 31, 51, 62, 76]. Of these 42 studies, 33 mentioned a decrease in glycated hemoglobin (HbA1c) [10,13,14,16,18,20,25,27,34,51–53,55–58,60–66,68–74,76,77,79], with a statistically significant reduction of HbA1c in 20 of them [13, 14, 20, 27, 34, 51, 53, 56, 60–65, 68, 70, 71, 74, 76, 79]. In one study [65], the reduction in HbA1c was observed only in the group of adolescents.

The relationship between the time of using CSII and the reduction in HbA1c was statistically significant in 18 studies: two of them described a reduction in the period from zero to three months of using CSII [62, 74]; four in the period of three to six months [51, 56, 61, 70]; eight in the period from six to twelve months [27, 53, 63–65, 71, 76, 79] and three in a period greater than 12 months [58, 60, 68]. The other studies did not describe the period for observing HbA1c reductions. Other factors that may influence the reduction in lower HbA1c values were also mentioned: greater parental involvement [70], time of diagnosis [70], frequency of glycemic monitoring [70, 80], high insulin sensitivity factor [80], low carbohydrate ratio value [80], greater amount of bolus insulin taken [80], younger children and adolescents [81], less intense autoimmune process [81] and lower HbA1c values in the first year of treatment [81].

In contrast, three studies showed that HbA1c did not change after using CSII [82–84]. One of these studies described a statistically significant reduction in HbA1c which occurred only in participants with worse glycemic controls [83]. Four studies showed that HbA1c increased after the use of CSII in some participants [61, 70, 85, 86]. These increases may be related to the adoption of inappropriate behaviors in the use of the CSII, such as the omission of bolus insulin [86], lower frequency of checking blood glucose [86], and age and time of diagnosis [70]. Some studies have reported both results, with an initial reduction in HbA1c and then a subsequent increase in this parameter; however, HbA1c remained at lower values when compared to previous use of CSII [12, 61, 70].

A study analyzed and compared the glycemic variability according to the CSII catheter puncture site, and found less variability when the catheter is inserted into the buttocks compared to the abdominal region placement [87]. One article demonstrated that each additional self-monitoring performed per day results in a decrease of approximately 0.2% in HbA1c [86]. The HbA1c values with the use of the CSII were also higher in a group of preschoolers compared to a group of adolescents [88]. Additionally, CSII has been used in two studies to induce insulin tolerance in patients with T1DM who are allergic to this substance, resulting in individualized treatment with good glycemic control [89, 90].

Nine studies concluded that the use of CSII reduced hypoglycemic episodes [17, 22, 49, 53, 55, 61, 70, 74, 84] and 11 reported that there was a reduction in severe hypoglycemic episodes [12, 13, 34, 49, 52, 64, 66, 71, 72, 75, 77]. Three studies described a lower occurrence of nocturnal hypoglycemic episodes [31, 37, 74] and another described a decrease in morning hypoglycemic episodes [89]. However, only eight studies demonstrated statistically significant hypoglycemic reductions [12, 22, 31, 61, 70, 71, 74, 84], of which one identified a reduction only in a group of children aged 10 to 12 years [64]. One of these articles is a literature review with inconclusive results regarding the increase or decrease in hypoglycemia rates [12]. Five studies showed a reduction in the risk of hypoglycemia [14, 25, 30, 36, 91], prevention of nocturnal hypoglycemia [28, 36] and the occurrence of this phenomenon in the morning [36].

Two studies reported a decrease in episodes of hyperglycemia after the use of CSII [57, 62]. However, two other articles described that such episodes can occur even when using CSII [23, 30]. According to studies, hyperglycemia can occur for different reasons, such as: blocking the insulin infusion due to cannula dislodgement [13, 21, 25, 30, 31, 78, 92, 93]; incorrect setting of basal rates or non-administration of bolus insulin [30]; the amount of insulin in the reservoir finishing [30]; battery running out [30]; and system occlusion [15, 33, 78, 83, 93, 94].

Eight studies described improvement in metabolic control with the use of CSII [10, 11, 19, 21, 33, 78, 94, 95]. The improvement in metabolic control was characterized by a reduction in plasma glucose [11]; circulating lipids [78]; plasma catecholamines [78]; cholesterol [21]; LDL, [21, 78]; anti-insulin antibodies [21]; amyloid protein A [21]; excessive losses of phosphorus and calcium in the urine [78]; and the concentration of glucose in the urine [10].

In three studies, a reduction in the frequency of diabetic ketoacidosis (DKA) episodes was mentioned after initiating the use of CSII [50, 66, 96], while two others reported that such episodes did not change compared to previous therapy [31, 61]. One study reported that episodes of DKA increased significantly after the use of CSII [79]. Thus, there is a controversy regarding a possible decrease or increase in episodes of DKA, as noted in a systematic review of the literature, which also did not present conclusive results about this increase or decrease in episodes of DKA [12].

In 13 studies, the risk of DKA, even when using CSII, was highlighted [13, 16, 17, 22, 23, 27, 30, 49, 52, 72, 92, 97, 98]. The occurrence of DKA episodes can be justified by the use of only fast-acting insulin in CSII [17]. Despite this, results from another study show that the longer the time of use of CSII, the lower the chances of DKA occurrence [99].

### Support networks

In this category the results of studies are gathered that contribute to the understanding of the necessary support for children and adolescents with T1DM in the use of CSII. The following subcategories were constructed: family support and support from the health team and the school, which will be detailed below.

#### Family support

Seven studies contributed to the construction of this subcategory [24, 33, 59, 69, 91, 94, 100]. Together, they showed that family support is important in the use of CSII by children and adolescents with T1DM. One article mentioned that parental support is necessary for the adjustment of insulin doses and also for the correction of any complications that may arise from the use of CSII [24], such as an eventual episode of severe hypoglycemia [78]. The experience of parents in caring for young children with the diagnosis of T1DM was described in a qualitative article, which presented reports of fear and insecurity at the beginning of the diagnosis, but being able to overcome these difficulties after using CSII [59].

Other aspects may influence the results, such as family support expressed by the level of parental involvement in handling and understanding the use of CSII [101], or in the transition of adolescent care in the management of T1DM [102]. In this sense, a study pointed out that in situations where parents handled their children’s CSII less frequently, children had worse glycemic control when compared to the children of parents who handled this more frequently [101]. Another important aspect to be highlighted is the support exercised by parents during childhood, ranging from comprehensive assistance between 3 and 5 years of age, shared management in the period of 6 to 12 years and support in the development of autonomy from 13 to 18 years [102, 103].

The need to offer family support and the type of family support to be offered can be interpreted differently by family members. In a study that evaluated the most challenging situations faced by parents and adolescents with T1DM, as well as the frequency of these situations, incongruity of perspectives was identified between adolescents and their parents [97]. According to the authors, the greatest difficulty in managing T1DM for adolescents involved self-care in social contexts and situations with their peers, while for parents it was related to situations that depended on the family context and other social contexts.

#### Support from the health team and the school

Fifteen studies showed the importance of the support of the health team in the care of children and adolescents with T1DM using CSII [16, 17, 22, 24, 33, 34, 37, 69, 72, 91, 94, 95, 100, 104, 105]. Of these, eight [17, 22, 69, 72, 91, 100, 104, 105] indicated that support can be favored in certain ways: through the provision of prior training given to the family and the patient at the beginning of the use of CSII [17, 22, 69, 72, 104]; detection by the health care team of the inappropriate use of CSII [100]; use of an educational program by the health team aiming to improve glycemic control [105]; identification of other health support, education, and knowledge networks; and development of skills necessary for behavior change [91].

One study highlighted that the health team needs to be trained to provide adequate support, care and management to patients using CSII [35]. Likewise, two studies showed that nurses who work in the school environment must be trained to provide the necessary care and support to children and adolescents who use CSII [37, 49]. In addition, it was also shown that nurses can provide support to those who are in out of the ordinary situations in the school context, such as dance presentations at school [49] and school trips [49, 57].

### Benefits of using CSII

The studies grouped together in this category show results that contribute to the understanding of the potential benefits of using CSII, with a view to the adequate management of T1DM. The benefits of using CSII found in this review are:

1. A more flexible lifestyle due to the use of CSII was addressed in 13 studies [13, 16, 22, 25, 27, 34, 37, 50, 54, 57, 106–108]. A more flexible lifestyle was mentioned by the studies including the possibility of traveling [106] and being able to fast due to religious traditions [50];
2. Fewer number of injections [14, 16, 17, 22, 23, 25, 34, 37, 109, 110], since the CSII remains connected for a period of 2 to 3 days, which eliminates the need for multiple daily injections [22].
3. The flexibility of meals was covered by 16 studies [13–15, 20, 23, 27, 28, 31–33, 36, 94, 106, 107, 109, 110]. The use of the CSII allows meals to be delayed, advanced or missed without compromising blood glucose control [27, 110]. The use of CSII can help normalize appetite, especially in young people with good glycemic control [107].
4. Improvement in the weight growth of children and adolescents [27, 95].
5. Improvement in eating behavior [109, 111], which resulted in better glycemic control [86].
6. Improvement of school performance and behavior [62, 112], such as performing tasks inside and outside the classroom environment [112].
7. Improvement of cognitive and technical skills [13, 15, 23, 24, 32, 103, 106]. Although some complex cognitive skills improve at the beginning of the use of CSII [32], a study pointed out that they must be taught by a trained health professional, and the adolescent must be able to demonstrate the skills with little difficulty [24].
8. Decrease in micro and macrovascular complications [17, 27, 33, 34, 49, 94]. Of these, one study [94] showed that the proper use of CSII reduces about 27-76% of micro and macrovascular complications and another study showed that the use of CSII reduces angiopathic and neuropathic complications in the long term [19]. One article highlighted the improvement in distal motor latency and the disappearance of painful disabling dysesthesia in an adolescent after 28 days of using CSII [73].
9. Reduction in hospital costs was presented by four studies [27, 57, 94, 96]. The evaluation of the use of the CSII resulted in a decrease in hospital costs, represented by the reduction in: the number of hospital admissions [57, 94]; costs with hospitalization [27, 94, 96]; and length of hospital stay [96].
10. Possibility of scheduling several doses of daily basal insulin [28, 34]. This is possible because, with CSII, the configuration of basal doses can vary according to the insulin requirement of the child and adolescent [34].
11. Improvement in quality of life and / or satisfaction with treatment [13, 16, 17, 23, 28, 32, 37, 52, 56, 57, 66, 69, 75, 77, 113]. One study [65] showed a significant improvement in quality of life related to T1DM after the transition to CSII, but there was no improvement in generic quality of life. Of the studies that showed improvement in the quality of life and / or satisfaction with the treatment, one [68] mentioned that 80% of the users of CSII were satisfied and happy with the treatment;
12. Possibility of storing the insulin dose records [17, 28, 77], since the CSII keeps the data of the administered insulin units stored in its history [17, 77].
13. Possibility of performing baseline and bolus doses with greater precision in the administration of low doses [15–17, 23, 37].
14. Possibility of programming the temporary basal insulin rates, which can be used on days of physical activity, menstruation and in the occurrence of illness [16, 17].
15. CSII has been described by three studies as being a safe and effective treatment [17, 22, 114].
16. Provides the desensitization of insulin resistance [17, 90], since it can be used even in cases of children and adolescents with T1DM who are allergic to insulin [90].

### Challenges of using CSII

The studies grouped together in this category show results that contribute to the understanding of the potential challenges of using CSII, with a view to the adequate management of T1DM. The challenges of using CSII found in this review were:

1. The complexity of using CSII has been described as a challenge in two studies [25, 59];
2. Eight studies highlighted the high economic cost of this technology [10, 14, 19, 20, 26, 37, 52, 115]. In addition, when calculating costs related to treatment with CSII, the time spent on intensive training for family members by the team of diabetes educators and doctors should be considered, in addition to the daily adjustments of insulin doses made by physicians [14];
3. The lack of adherence by both parents, children and adolescents in the treatment using CSII was mentioned in 14 studies as being a challenge [10, 36, 80, 114, 116–125]. Of these, seven showed that this lack of adherence directly interferes with the correct control of T1DM [80, 114, 116, 118, 120–122];
4. The need for developing complex skills in the use of CSII was highlighted in two articles [33, 94]. The mentioned difficulties include insertion of the catheter, skin care and inadequate monitoring;
5. Use of this technology in the school environment, as presented by a literature review [28];
6. Discomfort due to the need to be connected 24 h a day [25, 28, 30, 37];
7. Exclusive use of rapid insulin [28];
8. Body image problems [16, 28, 37, 49];
9. The need to increase insulin rates on sick days [30];
10. Disparities in access to the use of CSII [67, 115]. Patients using CSII with lower socioeconomic status are at higher risk of developing acute complications of T1DM, especially DKA [115]. One study identified less likelihood of using CSII in male, older, non-Hispanic blacks, indigenous young people, Alaskan Americans, Spanish speakers or non-English speakers, government insured or uninsured and patients with at least HbA1c ≥ 8.5% [67];
11. Possibility of error in the basal insulin schedules or in the administration of bolus doses [114].

In addition to the challenges mentioned above, other complications associated with the use of CSII were identified in the studies, namely: 1) needle rupture, mentioned in three studies [92, 126, 127]. Alternatively, a study recommended the use of a teflon catheter instead of steel needles [126]; 2) displacement of the cannula, discussed in eight studies [13, 21, 25, 30, 31, 37, 78, 93]; 3) infusion system occlusion, cited in seven studies as a complication [15, 33, 37, 78, 83, 93, 94]; 4) mechanical failures or system failures of the CSII, discussed in 16 studies [13, 21, 23, 25, 26, 30, 31, 52, 63, 69, 78, 93, 97, 98, 108, 128]; 5) Lipodystrophies [13, 93, 129]; 6) Infection at the CSII catheter insertion site was mentioned in 9 studies [13, 21, 26, 28, 30, 31, 69, 97, 130]; 7) Bleeding in the insertion and in the catheter was presented by one study [93]; and 8) Occurrence of dermatological problems such as allergies, irritations, eczema, among others reported by 12 studies [13, 21, 26, 30, 31, 37, 69, 97, 108, 129, 131, 132].

The discontinuity of treatment with CSII is also a challenge and was addressed in 13 studies [12, 20, 21, 31, 53, 58, 60, 65, 74, 83, 85, 115, 133]. Of these, 10 studies reported the number of children and adolescents who discontinued treatment, whose percentage varied between 0.42 and 19% of the total sample [12, 20, 31, 53, 58, 60, 65, 74, 85, 133]. The reasons why children and adolescents and their families supported each other in the decision to discontinue the use of CSII were: increased HbA1c [12, 85]; discrete results of blood glucose controls different to what was expected [53, 60, 85]; no improvement in the quality of life [53]; problems with the infusion set [53]; catheter obstruction [83]; occurrence of severe lipodystrophies [85]; body image problems [60]; and disadvantaged families followed up in small community centers [133].

Three studies [12, 20, 58] reported that the discontinuity in the use of CSII is greater in female patients [12, 20, 58], in pubertal age groups, with higher HbA1c, lower frequency of glycemic monitoring, from single-parent families and with higher rates of hypoglycemia [12].

## Discussion

This review grouped and described evidence available in the literature on the use of CSII in children and adolescents with T1DM, with regard to the metrics used for metabolic control; support networks; and benefits and challenges of using CSII. A rigorous mapping was also conducted of the literature on the use of CSII considering the location of development and the design of the studies [38, 39].

The results presented in this review were not conclusive in relation to the use of CSII on the reduction of HbA1c, reduction of episodes of hyperglycemia and DKA in children and adolescents with T1DM and, therefore, should be analyzed with caution. Although this technology has demonstrated success in the treatment of T1DM, the good outcomes in glycemic and metabolic control should not be analyzed in isolation.

As evidenced in this review, the metrics used for the glycemic and metabolic control of T1DM in children and adolescents are important indicators for the clinical follow-up of this population. However, the results highlight the lack of consensus between such metrics, and it is relevant to mention other aspects capable of influencing this control, such as socioeconomic and psychosocial factors [134]. These factors can be didactically presented as modifiable, for example, adherence to treatment, or non-modifiable, such as age, ethnicity and sex.

This review pointed out modifiable factors that interfere with the use of CSII, such as health literacy and the need for nutritional education. Similar results on the influence of these factors are described in the literature [135, 136]. A study carried out with young adults and adults identified that higher levels of education favored the improvement of glycemic control of T1DM, including patients using CSII [134]. Another factor, such as family structure, has also been described as an intervening factor in the glycemic control of children [137].

In addition to the modifiable factors, non-modifiable factors that influenced the use of CSII were also found in this review, these include racial and ethnic characteristics or people who are socially vulnerable [67]. Similar results were described in a retrospective study, in which black children and adolescents had higher levels of HbA1c and Caucasians more episodes of hypoglycemia [138]. Although some of these factors are not modifiable, it is important that health professionals know how to identify them and how to incorporate these characteristics in the design of educational approaches. For example, health care teams might consider implementing peer-mentorships [139] programs or group interventions [140] specific for underserved populations.

Other dimensions can also interfere in the good management of T1DM in children and adolescents using CSII, namely presence of support networks and family and health team support, including in the school environment. In this review, few studies were found that aimed to explore the influence of family support on the use of CSII. However, these studies were unanimous in describing the importance of family support both for handling CSII in unexpected situations resulting from T1DM and in the transition of care for adolescents with T1DM. Similar results indicate that the shared management of T1DM in adolescence reduces the risk of unfavorable outcomes [139].

The psycho-emotional aspects arising from the family dynamics described in this review can also interfere in the care of children and adolescents with T1DM using CSII. This results, for example, from parents’ difficulties and insecurities at the beginning of the diagnosis, from an ineffective ability to cope with T1DM, depression, and family conflicts, among others. Studies also report psycho-emotional repercussions in parents of children and adolescents with T1DM, including anxiety, depression [140], stress, fear of acute complications from T1DM, guilt over poor control, constant sadness resulting from situations experienced daily with their children, and risk of burnout [141].

The need to cope with the disease encourages parents to seek resources in support networks, in order to manage the exhaustion caused by the care of their children [141]. In this sense, it is essential to support health professionals in facing these psycho-emotional repercussions triggered by T1DM [140].

Support from the health team was represented in this review in the form of training, follow-up, use of play-based strategies and the identification of new support networks. The use of play-based strategies has been a primary tool for health professionals who deal with children and adolescents with T1DM, as it encourages self-care and good health practices [142, 143]. Such strategies can also be innovative for T1DM education using tools such as games, mobile apps and even humanoid robots [144, 145].

Few studies have addressed the support of the nursing staff or nurse educator of diabetes in the use of CSII. However, evidence points out that the inclusion of this professional in the care of patients with diabetes increases the regularity of medical appointments and, therefore, they tend to follow more carefully the health team’s recommendations for treatment [146–148].

The support of nurses or diabetes educators has been described as essential for the successful treatment of T1DM, especially with CSII [6, 7]. In addition, these professionals play an important role in onboarding and permanent training in the use of CSII, and also encourage the development of the seven self-care behaviors in T1DM, in accordance with the recommendations of the Association of Diabetes Care & Education Specialists and through prior training [149].

Finally, we highlight the identification of some challenges and benefits for the use of CSII. Currently, some of the challenges have already been overcome, one of which is the permanence of the subcutaneous puncture with the silicone catheter in place of the steel needle. Of the challenges described for the use of CSII that have not yet been overcome, there was a predominance of those related to the use of the CSII technological apparatus, followed by health literacy, family support and accessibility. Although these challenges have not yet been fully overcome, coping strategies are constantly being developed through public policies, in order to reduce the difficulty of accessing this resource [150–152]. It should be mentioned, as an example, the guarantee of access for use of CSII, a high cost device, to children and adolescents with T1DM through the judicialization of health, as might be the reality of lower or middle-income countries.

Nevertheless, multiple benefits were also identified in this review and support the indication of the use of CSII. Of these benefits, there is a predominance of those capable of promoting better quality of life for children and adolescents with T1DM, followed by the benefits related to behaviors, treatment facilities and health literacy. It is worth mentioning that both the challenges and the benefits can be experienced by some users of CSII and not by others, so that the experience of use is always subjective to each child and adolescent. Thus, the health professional must be updated and trained to clarify any doubts related to the challenges and benefits of therapy to family members and patients.

## Conclusion

This systematic mapping review made it possible to group and describe evidence of the main aspects of the use of CSII therapy for the management of T1DM in children and adolescents. Our findings clarify the treatment of T1DM with CSII, characterizing its importance in glycemic and metabolic control, the different supports needed for the success of the treatment, and its benefits and challenges. The use of this technology requires sufficient knowledge both from parents and caregivers as well as children and adolescents, self-care skills, and adherence to treatment. If the management of the aspects inherent to the use of the CSII in children and adolescents with T1DM is not sufficient for the safety of the patient and the achievement of the objectives established by the team, another type of treatment should be chosen that best suits the psychosocial and psycho-emotional conditions of the family. It is necessary that health professionals, including nurses, know and understand the particularities of this type of treatment for T1DM so that they can help the patient and family in the use of CSII. It is worth mentioning that both CSII and T1DM management remain in constant development.

## Supplementary Information

**Additional file 1.**

## Data Availability

Data sharing is not applicable to this article as no new data were created or analyzed in this study.

## Abbreviations

T1DM: Type 1 Diabetes Mellitus
CSII: Continuous Subcutaneous Insulin Infusion
T2DM: Type 2 Diabetes Mellitus
CGM: Continuous Glucose Monitoring System
UTM: Universal Transverse Mercator
SIRGAS: Geocentric Reference System for the Americas
HbA1c: Glycated hemoglobin
DKA: Diabetic Ketoacidosis
