# Supplementary material for: Use of continuous subcutaneous insulin infusion in children and adolescents with type 1 diabetes mellitus: a systematic mapping review
Source: BMC Endocr Disord. 2022 Feb 19;22:43. doi: 10.1186/s12902-022-00950-7 (PMC8858488; doi:10.1186/s12902-022-00950-7)
Supplement: Supplementary file 1 — Additional file 1. [file 12902_2022_950_MOESM1_ESM.pdf]

Population (P)<br><br>#1	“Child”[Mesh] OR (Child) OR (Children) OR “Adolescent”[Mesh] OR (Adolescent) OR (Adolescents) OR (Adolescence) OR (Teens) OR (Teen) OR (Teenagers) OR (Teenager) OR (Youth) OR (Youths)<br><br>**NOT**<br><br>(Adults) OR (Young adults)
#2	“Diabetes Mellitus, Type 1”[Mesh] OR (Diabetes Mellitus, Type I) OR (Type 1 Diabetes Mellitus) OR (Type 1 Diabetes) OR (Brittle Diabetes Mellitus) OR (Diabetes Mellitus, Brittle) OR (Diabetes Mellitus, Insulin-Dependent) OR (Diabetes Mellitus, Insulin Dependent) OR (Insulin-Dependent Diabetes Mellitus) OR (Diabetes Mellitus, Juvenile-Onset) OR (Diabetes Mellitus, Juvenile Onset) OR (Juvenile-Onset Diabetes) OR (Juvenile-Onset Diabetes Mellitus) OR (Diabetes Mellitus, Ketosis-Prone) OR (Diabetes Mellitus, Ketosis Prone) OR (Ketosis-Prone Diabetes Mellitus) OR (Juvenile-Onset Diabetes) OR (Juvenile Onset Diabetes) OR (Diabetes Mellitus, Sudden-Onset) OR (Diabetes Mellitus, Sudden Onset) OR (Insulin-Dependent Diabetes Mellitus 1) OR (Insulin Dependent Diabetes Mellitus 1) OR (IDDM) OR (Autoimmune Diabetes) OR (Diabetes, Autoimmune) OR (T1D)
#3	#1 AND #2
Context (C)<br><br>#4	"Insulin Infusion Systems"[Mesh] OR (Insulin Infusion Systems) OR (Insulin Infusion System) OR (infusion system, insulin) OR (infusion systems, insulin) OR (implantable programmable insulin pump) OR (pump, programmable implantable insulin) OR (insulin pump, programmable implantable) OR "Infusion Pumps"[Mesh] OR (Infusion Pumps) OR (Infusion Pump) OR (pump, infusion) OR (pumps, infusion) OR (perfusion pumps) OR (perfusion pump) OR (infusor) OR (drug infusion systems) OR (drug infusion system) OR (infusion systems, drug) OR (infusion system, drug) OR (system, drug infusion) OR (systems, drug infusion) OR (infusion pumps, external) OR (external infusion pump) OR (external infusion pumps) OR (infusion pump, external) OR (pump, external infusion) OR (pumps, external infusion) OR (Insulin pump)<br><br>**NOT**<br><br>(closed-loop glucose control) OR (closed-loop) OR (artificial pancreas) OR (hybrid closed-loop insulin delivery system) OR (closed-loop system)
Outcome (O)<br><br>#5	"Self-Management"[Mesh] OR (Self-Management) OR (self management) OR "Disease Management"[Mesh] OR (disease management) OR (management, disease) OR (managements, disease) OR "Knowledge Management"[Mesh] OR (Knowledge Management) OR (management, knowledge) OR (medication therapy management) OR (management, medication therapy) OR (therapy management, medication) OR (drug therapy management) OR (management, drug therapy) OR (therapy management, drug) OR "Self Care"[Mesh] OR (Self Care) OR (care, self) OR (self-care)
